# The positive feedback between ACSL4 expression and O-GlcNAcylation contributes to the growth and survival of hepatocellular carcinoma

**DOI:** 10.18632/aging.103092

**Published:** 2020-05-01

**Authors:** Jiachen Wang, Zhao Wang, Jiaxiang Yuan, Jiaxiang Wang, Xinsheng Shen

**Affiliations:** 1 Department of Minimally Invasive Surgery, The First Affiliated Hospital of Zhengzhou University, Zhengzhou 450052, Henan, China; 2 Department of Surgery, The First Affiliated Hospital of Zhengzhou University, Zhengzhou 450052, Henan, China

**Keywords:** ACSL4, O-GlcNAcylation, GLUT1, hepatocellular carcinoma

## Abstract

Acyl-CoA ligase 4 (ACSL4) has been reported to be overexpressed in hepatocellular carcinoma (HCC) and to enhance cell proliferation. However, the molecular mechanisms underlying the role of ACSL4 in HCC progression remain largely unclear. Here, we aimed to investigate whether and how O-GlcNAcylation and ACSL4 regulate each other and HCC progression. The clinical significance of ACSL4, O-GlcNAc and GLUT1 in HCC was determined by Pearson chi-squared test and Kaplan-Meier analysis. CCK-8, flow cytometry and *in vivo* tumour formation assays were performed to detect cell proliferation, apoptosis and tumorigenesis. IP technology was used to evaluate the relationship between ACSL4 and O-GlcNAc. ACSL4, GLUT1 and O-GlcNAc levels were elevated in HCC tissues and predicted poor prognosis in HCC patients. ACSL4 overexpression significantly promoted cell proliferation and tumorigenesis and inhibited cell apoptosis, whereas these effects were all obviously impaired when mTOR signalling was repressed or GLUT1 was downregulated. ACSL4 could be O-GlcNAcylated, and silencing of ACSL4 abolished the effects of O-GlcNAcylation on cell growth promotion and apoptosis inhibition. Collectively, this study demonstrates that ACSL4 contributes to the growth and survival of HCC by enhancing GLUT1-mediated O-GlcNAcylation. In turn, O-GlcNAcylation promotes HCC growth partially by increasing ACSL4 expression.

## INTRODUCTION

Hepatocellular carcinoma (HCC) is the second leading cause of cancer-related death among all malignant cancers worldwide, and its incidence is rising. Approximately 782,500 new liver cancer cases and 745,500 deaths were estimated in 2012 worldwide, with China alone accounting for approximately 50% [1, 2]. Although substantial progress has been made in the diagnosis and treatment of cancers, unfortunately, the prognosis of most HCC patients is still unsatisfactory [3].

It is well documented that deregulated cellular energetics is a characteristic hallmark of cancer cells [4], with an increased usage rate of glucose and glutamine, resulting in alterations in multiple metabolic and signalling pathways, including the hexosamine biosynthetic pathway (HBP) [5]. During glycolysis, enzymes of the HBP can transform fructose-6-phosphate into the end product UDP-N-acetylglucosamine (UDP-GlcNAc), which serves as the substrate for the O-linked N-acetylglucosamine (O-GlcNAc) modification of many nuclear and cytosolic proteins [5]. This post-translational modification is induced by O-GlcNAc transferase (OGT) and is removed by the O-GlcNAcase (OGA) [6, 7]. Recently, it was reported that O-GlcNAcylation can stabilize proteins directly or through competition with phosphorylation sites [7], thereby playing an important role in almost all cellular processes, such as growth, survival, apoptosis, migration, cycle and differentiation, as well as in the aetiology of many kinds of disease, including cancers [8–10]. In HCC, the O-GlcNAcylation of YAP was reported to confer YAP protein-enhanced tumorigenesis [11]. Additionally, O-GlcNAcylation can stabilize tribbles pseudokinase 2 (TRIB2) protein and thereby enhance its oncogenic role in liver cancer [12].

The acyl-CoA ligase 4 (*ACSL4*) gene that encodes the ACSL4 protein is a long-chain fatty acyl-CoA synthetase with a high specificity for arachidonic and eicosapentaenoic acid as substrates [13, 14]. Interestingly, intracellular ACSL4 protein content can be affected by free arachidonic acid [15]. Moreover, ACSL4 was reported to be overexpressed in HCC tissues and cells, and upregulation significantly enhanced the growth of HCC SNU 398 cells [16, 17], suggesting that ACSL4 exerts an oncogenic role in HCC. However, the underlying mechanism of ACSL4, such as whether ACSL4 can be O-GlcNAcylated or whether ACSL4 can regulate O-GlcNAcylation, remains largely unknown. As O-GlcNAcylation can enhance protein stability and thereby enhance protein function, we attempted to reveal the relationship between O-GlcNAc and ACSL4 in HCC in this study.

In addition, accumulated evidence demonstrates that the mammalian target of rapamycin (mTOR) signalling is a crucial event in the carcinogenesis of hepatocytes [18–21]. Works by Orlando et al. [22] indicated that ACSL4 was an activator of mTOR signalling, through which ACSL4 promoted the progression of breast cancer. However, whether mTOR signalling is involved in ACSL4-mediated HCC progression still needs to be elucidated.

In the present study, we aimed to determine whether and how O-GlcNAcylation and ACSL4 regulate each other and the progression of HCC. Our results demonstrated that ACSL4 could promote HCC growth and survival by enhancing O-GlcNAcylation and activating mTOR signalling. Conversely, GlcNAcylation facilitated HCC growth via increasing ACSL4 expression and activating mTOR signalling.

## RESULTS

### ACSL4 is highly expressed in HCC tissue samples and cells

**Figure 1:**
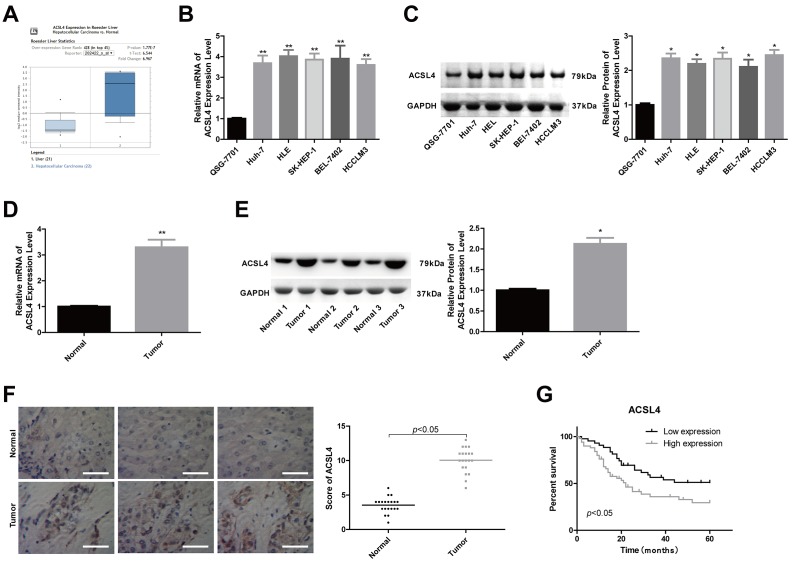
**ACSL4 was overexpressed in HCC tissue samples and cells.** (**A**) The Oncomine database was used to retrieve the different expression patterns of ACSL4 in HCC or normal liver. (**B**, **C**) The mRNA and protein content of ACSL4 in the human normal liver cell line QSG-7701 and the HCC cell lines Huh-7, HLE, SK-HEP-1, BEL-7402 and HCCLM3 were determined by RT-PCR and western blotting, respectively. (**D**, **E**) The mRNA and protein content of ACSL4 in HCC tissues and normal tissues were detected by RT-PCR and western blotting assays. (**F**) Immunohistochemistry was used to detect ACSL4 protein expression in HCC tissues and normal tissues (Scale bar = 100 μm). (**G**) Kaplan-Meier analysis of the relationship between ACSL4 expression and the overall survival of patients with HCC. (^*^P<0.05, ^**^P<0.01).

To explore the effects and reveal the underlying mechanism of ACSL4 in the progression of HCC, we first determined ACSL4 expression pattern in liver cancer tissues and cells. The Oncomine database showed that the expression of ACSL4 was significantly elevated in HCC samples (n=22) compared to normal liver samples (n=21) (Figure 1A). Consistently, compared with the human normal liver cell line QSG-7701, ACSL4 expression was significantly increased in HCC cell lines such as Huh-7, HLE, SK-HEP-1, BEL-7402 and HCCLM3 at both mRNA and protein levels (Figure 1B, 1C). Similarly, the increased expression of ACSL4 was also observed in HCC tissues compared with the adjacent normal liver tissues, which was determined by RT-PCR (Figure 1D), western blotting (Figure 1E) and immunohistochemistry (Figure 1F). These findings reveal that ACSL4 is overexpressed in HCC tissues and cell lines.

### High expression of ACSL4 predicts advanced clinical processes and poor outcomes in patients with HCC

Next, we explored the association between ACSL4 expression profiles and patient clinical features and prognosis after surgery in HCC patients. A total of 77 patients were included, among which 41 patients had high ACSL4 expression and 36 had low ACSL4 expression by immunohistochemistry staining. The results showed that patients with high ACSL4 expression were inclined to have shorter overall survival than those with low ACSL4 expression (Figure 1G). Moreover, the expression level of ACSL4 showed a positive correlation with tumour size (P=0.014), tumour amount, which refers to the number of metastatic tumours in locations such as the lung, pleura and brain (P=0.017), TNM stage (P=0.032), and the incidence of lymphonodus (P=0.042), embolus (P=0.022) and cirrhosis (P=0.02) (Table 1). These results suggest that the high expression level of ACSL4 is closely associated with advanced clinical processes and poor outcomes in HCC.

**Table 1:** Association between ACSL4 expressions with the clinical process of patients with HCC.

**Groups**	**Total cases**	**Low expression**	**High expression**	***P***
Gender				0.818
Male	48	25	23	
Female	29	16	13	
Age/years				0.228
<60	52	25	27	
≥60	25	16	9	
AFP/(μg/L)				0.067
<400	30	20	10	
≥400	47	21	26	
Size/cm				0.014
≤3	24	18	6	
>3	53	23	30	
Amount				0.017
1	59	36	23	
≥2	18	5	13	
Lymphonodus				0.042
Yes	21	7	14	
No	56	34	22	
Capsule				0.239
Yes	50	24	26	
No	27	17	10	
Embolus				0.022
Yes	16	4	12	
No	61	37	24	
TNM				0.032
I-II	49	31	18	
III-IV	28	10	18	
Cirrhosis				0.02
Yes	45	29	16	
No	32	12	20	

### ACSL4 promotes proliferation and inhibits apoptosis in HCC cells via activating mTOR signalling

**Figure 2:**
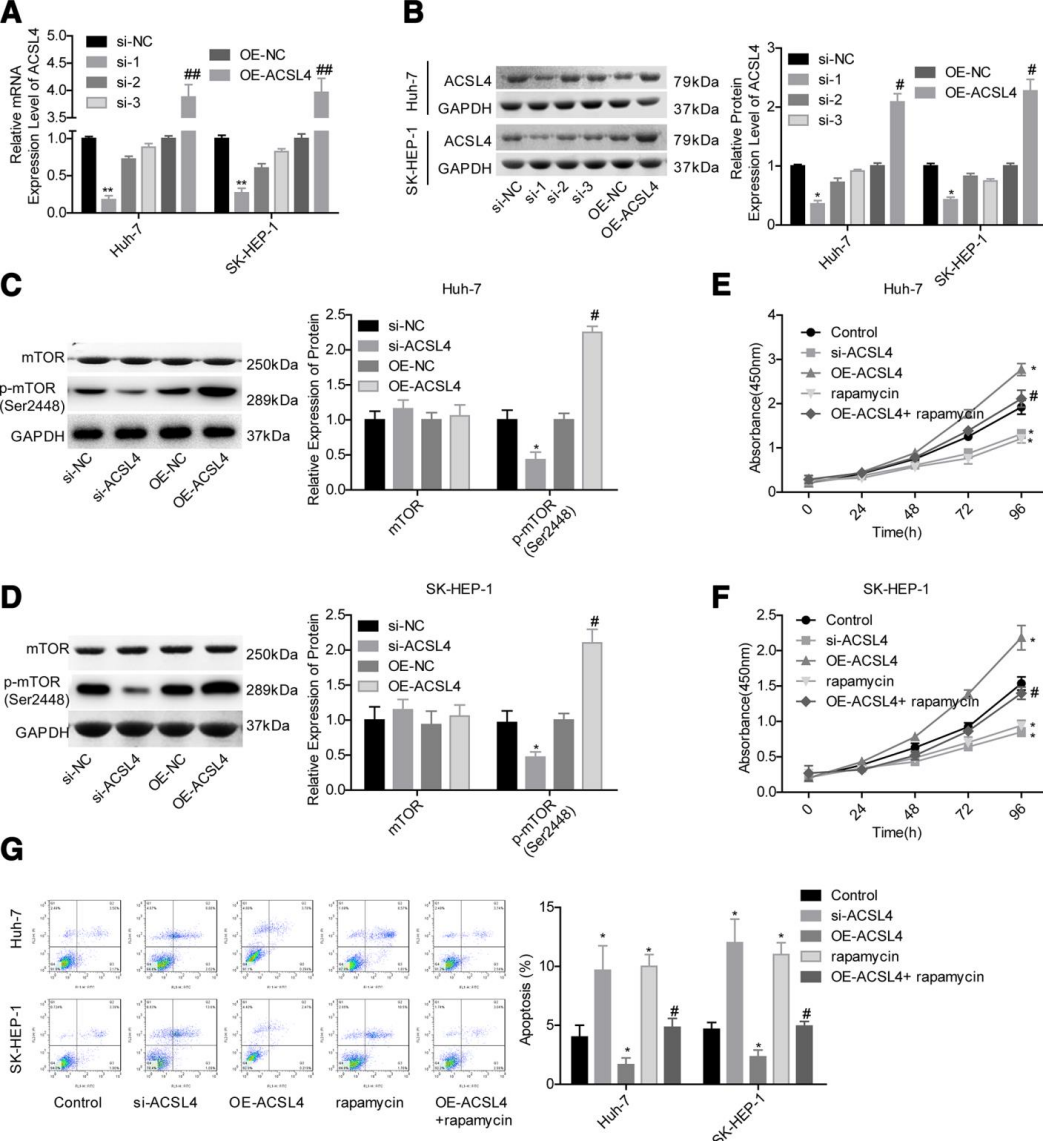
**ACSL4 promoted HCC cell proliferation and repressed cell apoptosis via activating mTOR signalling.** Huh-7 and SK-HEP-1 cells were transfected with si-NC, si-ACSL4, OE-NC or OE-ACSL4, with or without rapamycin treatment, and then the following assays were carried out. (**A**, **B**) RT-PCR and western blotting assays were carried out to assess the expression levels of ACSL4 at the mRNA and protein levels, respectively (^*^P<0.05, ^**^P<0.01, si-ACSL4 group compared with si-NC group; ^#^P<0.05, ^##^P<0.01, OE-ACSL4 group compared with OE-NC group). (**C**, **D**) The expression levels of mTOR and p-mTOR were detected by using a western blotting assay. (**E**, **F**) CCK-8 assay was used to detect cell proliferation (^*^P<0.05, compared with the control group; ^#^P<0.05, compared with the OE-ACSL4 group). (**G**) Flow cytometry assay was used to test cell apoptosis (^*^P<0.05, compared with the control group; ^#^P<0.05, compared with the OE-ACSL4 group).

Then, we carried out a gain/loss-of-function assay to assess the role of ACSL4 in HCC progression. Compared with the control group, the expression of ACSL4 was obviously decreased in the si-1 (siRNA-ACSL4-1) group, while it was significantly increased in the OE-ACSL4 (ACSL4 overexpression) group at the mRNA (Figure 2A) and protein levels (Figure 2B); hence, si-1 was chosen for further study. Then, we explored the effect of ACSL4 on the activation of mTOR signalling in HCC cells. The level of mTOR phosphorylation was obviously increased when ACSL4 was overexpressed, and it was reduced when ACSL4 was downregulated in both Huh-7 and SK-HEP-1 cell lines (Figure 2C, 2D). ACSL4 downregulation and rapamycin treatment significantly inhibited cell growth and induced cell apoptosis, while ectopic expression of ACSL4 caused the opposite results in both Huh-7 and SK-HEP-1 cells (Figure 2E–2G). Moreover, rapamycin treatment rescued the effects of ACSL4 overexpression in promoting cell growth and repressing cell apoptosis (Figure 2E–2G). These results illustrate that ACSL4 promotes HCC cell proliferation and represses cell apoptosis via activating mTOR signalling.

### ACSL4 plays a crucial role in O-GlcNAcylation-mediated HCC growth

**Figure 3:**
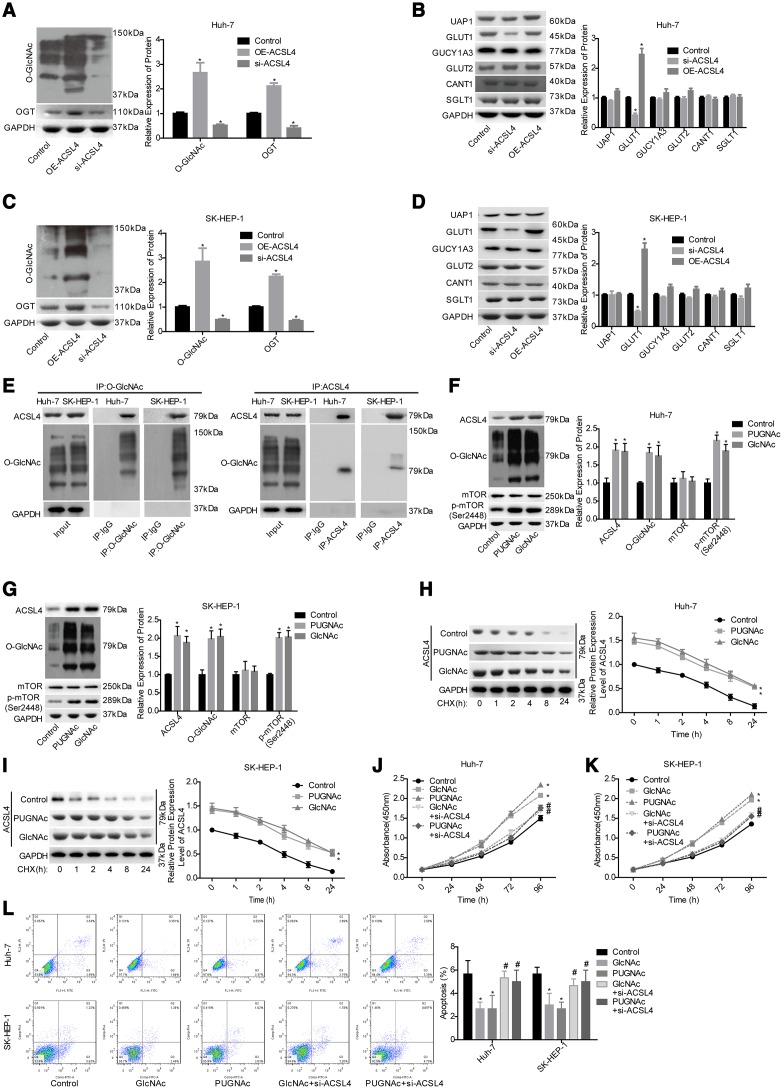
**Evaluation of the effects of ACSL4 on O-GlcNAc-mediated HCC growth.** Huh-7 and SK-HEP-1 cells were transfected with OE-ACSL4 or si-ACSL4, and then the cells were harvested for the western blotting assay to detect the expression of the following proteins. (**A**) OGT and O-GlcNAc in Huh-7 cells. (**B**) UAP1, GLUT1, GLUT2, GUCY1A3, CANT1 and SGLT1 in Huh-7 cells. (**C**, **D**) OGT, O-GlcNAc, UAP1, GLUT1, GLUT2, GUCY1A3, CANT1 and SGLT1 in SK-HEP-1 cells. (**E**) IP assay used to detect the interaction between O-GlcNAc and ACSL4 with an antibody against O-GlcNAc or ACSL4. IgG served as a negative control. Then, the si-ACSL4-transfected or untransfected Huh-7 and SK-HEP-1 cells were treated with PUGNAc, GlcNAc or nothing, and the following assays were carried out. (**F**, **G**) The levels of ACSL4, O-GlcNAc, mTOR and p-mTOR were determined by using a western blotting assay. (**H**, **I**) The protein stability was determined by western blotting after incubation with CHX (100 μg/ml) for 0, 1, 2, 4, 8 or 24 hours. (**J**, **K**) Cell proliferation was detected by CCK-8 assay. (**L**) Cell apoptosis was assessed by flow cytometry assay. (**A**–**D**) ^*^P<0.05, si-ACSL4/OE-ACSL4 group compared with control group; (**E**–**L**)^*^P<0.05, PUGNAc/GlcNAc group compared with control group; ^#^P<0.05, PUGNAc + si-ACSL4/GlcNAc + si-ACSL4 group compared with PUGNAc/GlcNAc group).

Next, we explored the effect of ACSL4 on the O-GlcNAcylation level in HCC cells. Compared with the control group, the OE-ACSL4 group showed higher levels of OGT and O-GlcNAc, while the si-ACSL4 group showed decreased levels of OGT and O-GlcNAc in both Huh-7 (Figure 3A) and SK-HEP-1 cell lines (Figure 3C). In addition, we explored the effects of ACSL4 on the expression of main proteins in glycosylated signalling, including UAP1, GUCY1A3, GLUT1, GLUT2, CANT1 and SGLT1 [23]. The results showed that only the expression of GLUT1 was significantly increased after ACSL4 was overexpressed in Huh-7 and SK-HEP-1 cells, with no obvious change in the expression levels of UAP1, GUCY1A3, GLUT2, CANT1 and SGLT1 (Figure 3B, 3D). Subsequently, we explored the relationship between ACSL4 expression and O-GlcNAc and their roles in HCC progression. The immunoprecipitation (IP) assay with either anti-ACSL4 or anti-O-GlcNAc antibody demonstrated that ACSL4 protein could be O-GlcNAcylated (Figure 3E). In addition, the enhanced O-GlcNAcylation level induced by GlcNAc and PUGNAc treatment significantly increased the expression of ACSL4 and p-mTOR (Figure 3F, 3G) and increased the protein stability of ACSL4 (Figure 3H, 3I). Furthermore, cell proliferation was significantly enhanced and cell apoptosis was reduced when the cells were treated with either GlcNAc or PUGNAc (Figure 3J–3L). However, these effects were all significantly weakened when ACSL4 was downregulated in both Huh-7 and SK-HEP-1 cell lines (Figure 3J–3L). These results suggest that O-GlcNAcylation promotes HCC cell growth partially via increasing ACSL4 expression.

### ACSL4 promotes HCC growth in a GLUT1-dependent way

**Figure 4:**
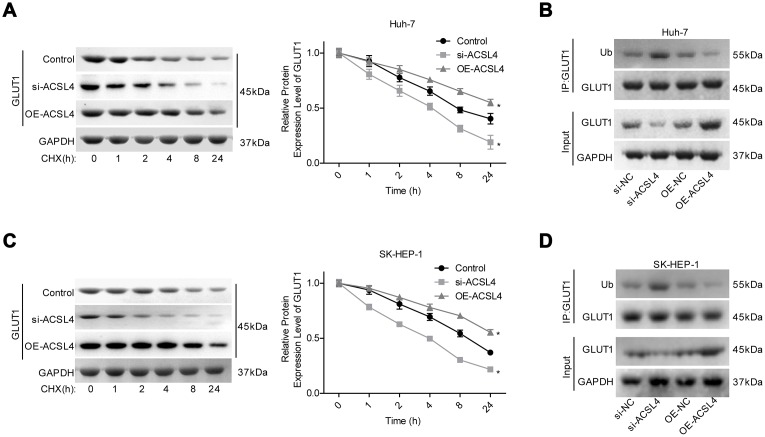
**ACSL4 upregulation enhanced the stability of GLUT1 protein and reduced its ubiquitination.** (**A**) After 12 hours of cell transfection with si-ACSL4 or OE-ACSL4, Huh-7 cells were treated with CHX (100 μg/ml) for 0, 1, 2, 4, 8 or 24 hours, and the western blotting assay was performed to detect GLUT1 expression. (**B**) An IP assay was used to detect the interaction between Ub and GLUT1 proteins after Huh-7 cells were transfected with si-ACSL4 or OE-ACSL4. (**C**) After 12 hours of cell transfection with si-ACSL4 or OE-ACSL4, SK-HEP-1 cells were treated with CHX (100 μg/ml) for 0, 1, 2, 4, 8 or 24 hours, and the western blotting assay was performed to detect GLUT1 expression. (**D**) IP assay was used to detect the interaction between Ub and GLUT1 protein in SK-HEP-1 cells. (^*^P<0.05, si-ACSL4/OE-ACSL4 group compared with control group).

**Figure 5:**
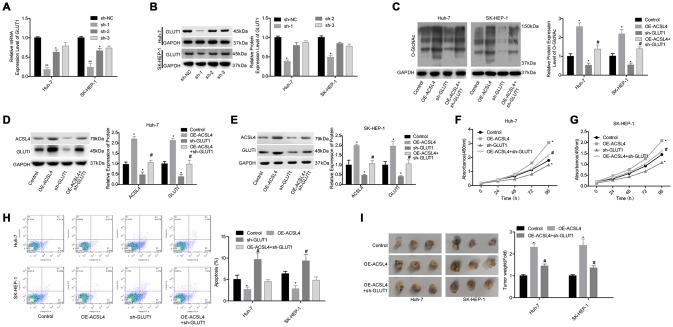
**Evaluation of the effects of the ACSL4/GLUT1 axis on cell proliferation, apoptosis and tumorigenesis in Huh-7 and SK-HEP-1 cells.** (**A**, **B**) The mRNA and protein expression levels of GLUT1 were determined by RT-PCR and western blotting assays after cells were transfected with sh-GLUT1 or sh-NC, respectively (^*^P<0.05, ^**^P<0.01, compared with the sh-NC group). Next, Huh-7 and SK-HEP-1 cells were transfected with OE-ACSL4 and/or sh-GLUT1 and subjected to the following assays. (**C**–**E**) Western blotting assays were used to assess the levels of O-GlcNAc, ACSL4 and GLUT1. (**F**, **G**) CCK-8 assay was carried out to test cell proliferation. (**H**) Flow cytometry assay was used to determine cell apoptosis. (**I**) An *in vivo* xenotransplantation assay was used to assess the effects of the ACSL4/GLUT1 axis on the tumour formation ability of Huh-7 and SK-HEP-1 cells. (**C**–**I**: ^*^P<0.05, compared with control group; ^#^P<0.05, compared with OE-ACSL4 group).

Next, we explored whether GLUT1 was involved in ACSL4-mediated promotion of HCC cell growth. ACSL4 upregulation significantly slowed the protein degradation of GLUT1 (Figure 4A, 4C) and reduced GLUT1 ubiquitination (Figure 4B, 4D). Transfection of si-1 (siRNA-1) targeting the human GLUT1 gene significantly reduced GLUT1 expression at both the mRNA and protein levels (Figure 5A, 5B). In addition, the results of the western blotting assay showed that ACSL4 upregulation apparently increased the expression of O-GlcNAc and GLUT1, but this effect was abolished when GLUT1 was silenced in Huh-7 and SK-HEP-1 cells (Figure 5C–5E). The CCK-8 and flow cytometry results showed that cell growth promotion and apoptosis repression induced by ACSL4 were neutralized by GLUT1 downregulation (Figure 5F–5H); the tumorigenesis induced by ACSL4 overexpression was also attenuated, as detected by the *in vivo* animal assay (Figure 5I). These results demonstrate that GLUT1 is strongly implicated in ACSL4-mediated HCC growth.

### Evaluation of the clinical significance of GLUT1 and O-GlcNAc in HCC

**Figure 6:**
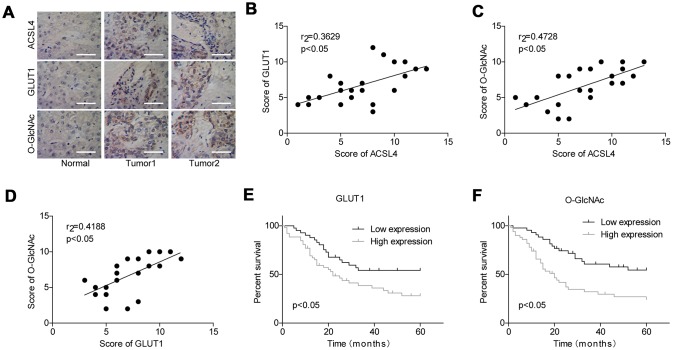
**Evaluation of the levels of GLUT1 and O-GlcNAc in HCC and their clinical significance.** (**A**) Immunohistochemistry technology was used to assess the protein levels of ACSL4, O-GlcNAc and GLUT1 in HCC tissues and adjacent normal tissues (Scale bar = 100 μm). (**B**–**D**) Pearson correlation analysis of the correlations between the levels of ACSL4, O-GlcNAc and GLUT1 in HCC tissues. (**E**, **F**) Kaplan-Meier analysis of the relationship between GLUT1/O-GlcNAc levels and the overall survival of patients with HCC.

Finally, we assessed the association between the expression patterns of O-GlcNAc, ACSL4 and GLUT1 in HCC. As shown in Figure 6A, the protein levels of O-GlcNAc, ACSL4 and GLUT1 were all elevated in tumour tissues compared with adjacent normal tissues. The expression levels O-GlcNAc, ACSL4 and GLUT1 were all positivity correlated with one another (Figure 6B–6D). Moreover, similar to the significance of ACSL4 level in predicting patient overall survival, patients with high expression of GLUT1 (Figure 6E) or O-GlcNAc (Figure 6F) always had shorter overall survival than those with low expression levels of GLUT1 or O-GlcNAc. These results illustrate that the high expression of GLUT1 and O-GlcNAc predicted a poor prognosis in patients with HCC.

## DISCUSSION

Fatty acids are essential nutrients that play a vital role in maintaining physiological function via energy metabolism and cellular signalling pathways. The deregulation of fatty acid metabolism contributes to excess lipid biosynthesis and deposition, which eventually leads to the body with metabolic disorders, even cancer initiation and development [24]. Long-chain acyl-CoA synthetase (ACSL) enzymes are essential for the activation of the most abundant long-chain fatty acids, which contain 12-20 carbons [25, 26]. In the present study, we focused on the function of ACSL4, a member of the ACSL family, in the progression of HCC and its underlying mechanisms. Our results demonstrated the potential value of ACSL4 as a potential biomarker and therapeutic target for HCC.

Consistent with previous findings [16, 17], we found that ACSL4 was highly expressed in HCC tissues and cell lines, such as Huh-7, HLE, SK-HEP-1, BEL-7402 and HCCLM3, compared to normal controls. Moreover, we observed that high expression level of ACSL4 predicted malignant clinical features and poor prognosis in HCC patients after surgery. Similarly, the works by Sun et al. [27] also revealed that high ACSL4 expression levels were significantly associated with Edmondson grade (p=0.010), AFP (p=0.001) and TNM stage (p=0.012), overall survival (p=0.001) and disease-free survival (DFS) (P=0.000) in HCC patients. All of the findings confirm the vital clinical role of ACSL4 in HCC.

Accumulating evidence has highlighted the vital role of ACSL4 in regulating cancer cell proliferation and apoptosis. Downregulation of ACSL4 induced by the synergistic therapy of aspirin and sorafenib significantly weakened cell tumorigenesis and induced cell apoptosis in HCC Hep3B and HuH-7 cells [28]. By contrast, increased expression of ACSL4 promoted an invasive phenotype in oestrogen receptor-positive mammary carcinoma cells [29] and increased cell proliferation and tumour formation abilities [30–32]. Additionally, inducible expression of ACSL4 could significantly rescue the enhancement of cell apoptosis induced by arachidonic acid accumulation through esterification of arachidonic acid into cellular triacylglycerol (TAG) [33]. Coincidently, in the present study, we also revealed that ACSL4 promotes HCC via increasing cell proliferation and *in vivo* tumour growth and reducing cell apoptosis. Furthermore, our results also elucidated that ACSL4 functions as an activator of mTOR signalling, through which ACSL4 increases HCC cell growth and represses cell apoptosis. ACSL4 acts by this same mechanism in breast cancer [22].

As elevated O-GlcNAcylation has been reported to play a vital role in HCC progression [34, 35], we assessed the relationship between O-GlcNAcylation and ACSL4. We observed that ACSL4 overexpression significantly increased the O-GlcNAcylation level. In addition, the expression levels of HBP components, GUCY1A3, UAP1 and CANT1, and three main glucose transporters, GLUT1, GLUT2 and SGLT1 [36, 37] were detected by western blotting to explore the possible mechanism underlying ACSL4-mediated O-GlcNAcylation enhancement. We observed that the altered expression of ACSL4 only affected GLUT1 expression significantly and that it showed no obvious effect on other HBP components or glucose transporters. GLUT1, also known as SLC2A1, is a member of the integral membrane glucose transporter family, which is essential for the transportation of glucose and other carbohydrates into cells [38]. GLUT1 is reported to be modestly expressed in most cells, with the highest expression in the blood brain barrier, erythrocytes, eye, placenta, neuronal membranes and lactating mammary glands [39, 40]. Aside from its developmental role [41], GLUT1 has been identified to be deregulated in a variety of cancers [42–44], and is reported to promote the aggressive progression of gastric cancer [45]. Moreover, GLUT1 was found to be positively expressed in 24/100 HCC tissues and 0/100 adjacent normal tissues and was indicated to promote HCC progression via the positive regulation of forkhead box M1 (FOXM1) [46]. In the present study, we demonstrated that the GLUT1 and O-GlcNAc levels were significantly increased in HCC tissues and were closely related to a poor prognosis in patients with HCC. Furthermore, we demonstrated that GLUT1 was an important intermediate component in ACSL4-induced O-GlcNAcylation, HCC cell proliferation, tumorigenesis and apoptosis inhibition, suggesting that ACSL4 promoted HCC growth via GLUT1-mediated O-GlcNAcylation elevation.

In a previous study, the O-GlcNAcylation of c-Jun and TRIB2 was reported to promote liver tumourigenesis [12, 47]. Here, we not only demonstrated that ACSL4 protein could be O-GlcNAcylated but also explored whether ACSL4 was involved in O-GlcNAcylation-mediated HCC growth. We found that the increased level of O-GlcNAcylation induced by GlcNAc or PUGNAc treatment resulted in significant increases in both ACSL4 expression and protein stability. In turn, we demonstrated that ACSL4 was essential for O-GlcNAcylation to promote HCC growth, which further extends the cognition of O-GlcNAcylation in accelerating liver tumourigenesis.

In conclusion, the present study demonstrates that ACSL4 contributes to HCC growth and survival via enhancing GLUT1-mediated O-GlcNAcylation. In turn, O-GlcNAcylation facilitates HCC growth partially via increasing ACSL4 expression. Our study provides a new mechanism by which O-GlcNAcylation and ACSL4 accelerate HCC growth, which might serve as an efficient target for HCC treatment.

## MATERIALS AND METHODS

### Bioinformatics analysis

The Oncomine database (https://www.oncomine.org/resource/login.html) was used to find the different expression patterns of ACSL4 in HCC tissues and normal liver tissues.

### Liver tissue samples

A total of 77 paired HCC tissues and their matched normal liver tissues were obtained from patients with HCC who underwent hepatectomy prior to radiotherapy or chemotherapy. All patients provided informed consent form. The protocols involving human samples were performed in accordance with the Helsinki Declaration and were approved by the ethical committee of the First Affiliated Hospital of Zhengzhou University. The tumour amount refers to the number of metastatic tumours, such as lung, pleura and brain, which was measured by X-ray or CT (computerized tomography).

### Immunohistochemistry

Immunohistochemistry assays were carried out to detect ACSL4 expression patterns in HCC tissues and paired normal tissues according to the manufacture protocol. Briefly, after dewaxing, antigen repair, and serum sealing with 5% goat serum (Solarbio, Beijing, China), the 4-μm sections were incubated with anti-ACSL4 antibody (1: 100 dilution, no. ab110007, Abcam, MA, USA), anti-O-GlcNAc antibody (1:200 dilution; no. MA1-072, Thermo Fisher Scientific, MA, USA) or anti-GLUT1 (GLUT) antibody (1:200 dilution; no. PA5-16793, Thermo Fisher Technology) at 4 °C overnight. The next day, the sections were incubated with the corresponding second antibody (Zhongshanjinqiao, Beijing, China) at room temperature for 1 hour. The staining results were evaluated by using a microscope, and the immunohistochemical scores were calculated by two researchers who were blinded to the grouping information. For staining extent, positive cells <5% were scored as 0, positive cells 5%-25% were scored as score 1, positive cells 25%-50% were scored as score 2, and positive cells >75% were scored as score 3. For staining intensity, 0, 1, 2 and 3 represent no, weak, intermediate and strong staining, respectively. The staining extent score and intensity score were multiplied to obtain the final score.

### Cell lines and culture

The human normal liver cell line QSG-7701 and the HCC cell lines Huh-7, HLE, SK-HEP-1, BEL-7402 and HCCLM3 were purchased from the American Type Culture Collection (ATCC, Manassas, VA, USA). QSG-7701, HLE and BEI-7402 cells were maintained in RPMI-1640 medium, Huh-7 and HCCLM3 cells in Dulbecco’s modified Eagle’s medium with high glucose (DMEM-H), and SK-HEP-1 cells in modified Eagle’s medium (MEM), all supplemented with 1% foetal bovine serum (FBS) and 1% penicillin/streptomycin (v/v). All regents used in cell culture were obtained from Thermo Fisher Scientific (MA, USA).

### Cell treatments

To enhance O-GlcNAcylation, Huh-7 cells were treated with 75 μM O-(2-acetamido-2-deoxy-D-glucopyranosylidene) amino-N-phenylcarbamate (PUGNAc), an inhibitor of OGA or 5 mM glucosamine (GlcNAc) for 24 hours at 37 °C. Cycloheximide (CHX) was used to stop protein synthesis at a final concentration of 100 μg/ml for 1, 2, 4, 8 or 24 hours. To repress the activation of mTOR signalling, the cells were treated with 10 nM rapamycin (MedChemExpress, USA) for 24 hours.

### Alteration of gene expression in HCC cells

Small interfering RNAs (siRNAs) targeting the human ACSL4 gene (si-ACSL4) were purchased from Origene (Beijing, China; no. SR301523) and were transfected into HCC cells to downregulate ACSL4 expression. The overexpressing (OE) lentivirus vector of ACSL4 (OE-ACSL4) and its negative control (OE-NC) were purchased from Origene (no. RC221413L4) and were used to upregulate ACSL4 expression. The short hairpin RNAs (shRNAs) of GLUT1 (sh-GLUT1) used to downregulate GLUT1 was synthesized by GenePharma (Shanghai, China).

### Real-time PCR (RT-PCR)

Total RNA was extracted from tissues and cells using the RNAsimple total RNA extract kit (no. DP419, TIANGEN, Beijing, China). Then, a total of 1 μg RNA was reverse transcribed to cDNA by using a First Strand cDNA Synthesis Kit (CWBIO, Jiangsu, China), followed by RT-PCR performance on an ABI PRISM 7700 Sequence Detection System (Applied Biosystems, Foster City, CA, USA) with SYBR Green (CWBIO) in a 20-μl reaction system. GAPDH was used as an internal reference to normalize mRNA expression. Primer sequences are listed as follows:

ACSL4: sense, 5’-CGGTTCCTTTTTGCGAGCTT-3’,

ACSL4: antisense, 5’-AAAGTACGCAAATGTCCTCTTTT-3’;

GLUT1: sense, 5’-TGAGCATCGTGGCCATCTTT-3’,

GLUT1: antisense, 5’-CCGGAAGCGATCTCATCGAA-3’;

GAPDH: sense, 5’-CCACTAGGCGCTCACTGTTCT-3’,

GAPDH: antisense, 5’-GCATCGCCCCACTTGATTTT-3’.

### Western blotting assay

Western blotting assays were performed to detect protein expression. RIPA lysis buffer (Beyotime, Jiangsu, China) was used to extract the total protein from tissue samples and cells. After quantification with a BCA kit (Thermo Fisher Scientific) and degeneration at 100 °C for 10 min, 25 μg protein from each sample was loaded onto 10% SDS-PAGE and then transferred into PVDF membranes (Thermo Fisher Scientific), followed by immunoblotting by incubating with the indicated primary antibodies overnight at 4 °C. Then, the membranes were incubated with the corresponding secondary antibodies (Zhongshanjinqiao) for 1 hour at room temperature. The blot bands were visualized by an enhanced chemiluminescence regent (ECL; Millipore) and detected on the gel imaging instrument (Eberhardzell, Germany). ImageJ software was used to quantify protein expression. Primary antibodies are as follows: ACSL4 (no. ab110007, Abcam), O-GlcNAc (no. MA1-072, Thermo Fisher Scientific), OGT (1:1000 dilution; no. 24083, Cell Signaling Technology, CA, USA), UAP1 (no. HPA014659, Merck, Darmstadt, Germany), GLUT1 (no. PA5-16793, Thermo Fisher Technology), GLUT2 (no. ab54460, Abcam), GUCY1A3 (guanylate cyclase 1 soluble subunit alpha 1) (no. 12605-1-AP, Wuhan, China), CANT1 (calcium activated nucleotidase 1) (no. HPA019639, Merck), SGLT1 (solute carrier family 5 member 1) (no. ab14686, Abcam), mTOR (no. 2972, Cell Signaling Technology), p-mTOR (no. 5536, Cell Signaling Technology) and GAPDH (1:5000 dilution; no. 5174, Cell Signalling Technology).

### Immunoprecipitation (IP)

The IP assay was performed as previously described [48] to assess the interactions between proteins. In detail, Huh-7 cells were collected and lysed in Western/IP lysis buffer (Beyotime, Jiangsu, China) according to the manufacturer’s description. After preclearing with 50 μl of protein A/G-Sepharose (Novex, Oslo, Norway) for 1 hour, the supernatants were incubated overnight at 4 °C with anti-O-GlcNAc (no. 9875, Cell Signaling Technology), anti-ACSL4 antibody (no. ab110007, Abcam) or IgG for crosslinking and then incubated with protein A/G-Sepharose beads and washed with the Western/IP lysis buffer (Beyotime). Then, the beads were subjected to western blotting assay with the indicated antibodies.

### CCK-8 (cell counting kit-8) assay

The CCK-8 Kit (MedChemExpress, Shanghai, China) was used to assess cell proliferation. In detail, transfected or untransfected Huh-7 or SK-HEP-1 cells during the logarithmic growth phase were collected to prepare cell suspensions. Next, 100 μl of cell suspension containing 2000 cells was added into each well of the 96-well plates. Then, 10 μL of CCK-8 solution was added into each well after 24, 48, 72 and 96 hours of cell inoculation. OD values were measured at 450 nm with a microplate reader after another 4 hours of incubation.

### Flow cytometry assay

The Annexin V (FITC) Apoptosis Detection Kit purchased from BD Biosciences (San Diego, CA, USA) was recruited to determine cell apoptosis. After 48 hours of cell treatment, Huh-7 or SK-HEP-1 cells were collected with 0.25% EDTA-free trypsin (Thermo Fisher Scientific) and resuspended in 100 μl of 1X binding buffer solution containing 5 μl of annexin V and 5 μl PI of solution and incubated in the dark for 15 min. Subsequently, the cells were washed with 1X binding buffer and resuspended in 500 μl of 1X binding buffer. Cell apoptosis was measured with a Beckman FC500 flow cytometer (Beckman Coulter, Inc., Brea, California, USA) and analysed with FlowJo 7.6 software.

### Xenotransplantation

Protocols involving animals were approved by the Animal Care and Use Committee of the First Affiliated Hospital of Zhengzhou University. Huh-7 and SK-HEP-1 cells were stably transfected with OE-ACSL4 or OE-ACSL4 + sh-GLUT1 induced by selecting 7 μg/ml puromycin and 7 μg/ml puromycin + 100 μg/ml G418 for 14 days. Then, the cells (5×10^6^) were injected into the armpit area of 6-week-old nude mice (Beijing Vital River Laboratory Animal Technology Co., Ltd., Beijing, China), 5 mice/group. Twenty-eight days post-transplantation, mice were sacrificed, and the tumours were collected for weighing.

### Statistical analysis

Each experiment in the present study was performed at least three times, and the data are expressed as the mean ± square deviation (SD). SPSS 17.0 software was used for statistical analysis. In detail, Student’s t test or one-way ANOVA test was applied to perform data analysis between 2 groups or multiple groups, respectively. Pearson chi-squared test was used to determine the clinical significance of ACSL4 expression in patients with HCC. Kaplan-Meier analysis was used to analyse the parameters associated with the overall survival of HCC patients. A P value <0.05 was considered statistically significant.
